# Highly Sensitive Qualitative and Quantitative Identification of Cashmere and Wool Based on Terahertz Electromagnetically Induced Transparent Metasurface Biosensor

**DOI:** 10.3390/bios14050240

**Published:** 2024-05-10

**Authors:** Dongpeng Luo, Limin Xu, Lifeng Jia, Lianglun Cheng, Ping Tang, Jinyun Zhou

**Affiliations:** 1 School of Physics and Optoelectronic Engineering, Guangdong University of Technology, Guangzhou 510006, China; 2112115121@mail2.gdut.edu.cn; 2 Key Laboratory of Photonics Technology for Integrated Sensing and Communication of Ministry of Education, Guangdong University of Technology, Guangzhou 510006, China; xulimin@gdut.edu.cn; 3 School of Information Engineering, Guangdong University of Technology, Guangzhou 510006, China; 4 School of Computer Science and Technology, Guangdong University of Technology, Guangzhou 510006, China; lfjia@gdut.edu.cn; 5 School of Art and Design, Guangdong University of Technology, Guangzhou 510006, China; llcheng@gdut.edu.cn

**Keywords:** cashmere and wool, terahertz metasurface biosensor, electromagnetic inductive transparency, qualitative and quantitative

## Abstract

Cashmere and wool are both natural animal fibers used in the textile industry, but cashmere is of superior quality, is rarer, and more precious. It is therefore important to distinguish the two fibers accurately and effectively. However, challenges due to their similar appearance, morphology, and physical and chemical properties remain. Herein, a terahertz electromagnetic inductive transparency (EIT) metasurface biosensor is introduced for qualitative and quantitative identification of cashmere and wool. The periodic unit structure of the metasurface consists of four rotationally symmetric resonators and two cross−arranged metal secants to form toroidal dipoles and electric dipoles, respectively, so that its effective sensing area can be greatly improved by 1075% compared to the traditional dipole mode, and the sensitivity will be up to 342 GHz/RIU. The amplitude and frequency shift changes of the terahertz transmission spectra caused by the different refractive indices of cashmere/wool can achieve highly sensitive label−free qualitative and quantitative identification of both. The experimental results show that the terahertz metasurface biosensor can work at a concentration of 0.02 mg/mL. It provides a new way to achieve high sensitivity, precision, and trace detection of cashmere/wool, and would be a valuable application for the cashmere industry.

## 1. Introduction

Cashmere and wool are natural animal fibers and important raw materials in the textile industry [1]. However, cashmere is rarely produced compared to wool, and cashmere is lighter, softer, and warmer than wool, thus these make the price of cashmere tens of times the price of wool. With the gradual increase in demand for cashmere products, the global cashmere apparel industry was estimated to be worth $3015.98 million in 2021 and is expected to grow to $4105.41 million by 2029, at a CAGR (Compound Annual Growth Rate) of 3.93% from 2022 to 2029 [2]. Adulteration in cashmere textiles due to the presence of wool counterfeiting as cashmere for a lower cost has made the qualitative and quantitative detection of cashmere and wool fibers increasingly necessary [3,4].

Cashmere and wool are traditionally identified using microscopic [5], DNA [6,7,8], chemical [9], proteomic, and computer vision (based on image processing) methods [10,11,12]. However, each method has its shortcomings in practical application. For example, microscopic identification is only suitable for the identification of undyed and undamaged wool and cashmere. Furthermore, the use of microscopic analysis to accurately identify cashmere and wool requires a high level of professionalism on the part of the inspector. DNA analysis is a reliable identification technique, but it requires very expensive equipment and a complex testing process that must be carried out by specialized personnel. Chemical treatment of cashmere and wool with same solution makes cashmere more prone to stretching than wool, but the chemical method is only suitable for the qualitative identification of cashmere and wool, though it carries risks. The proteomic analysis method uses the proteins contained in the fibers to identify the fibers, which is used more for the qualitative detection of fibers and requires further research into quantitative detection. It is also difficult to perform quantitative analysis using computer vision−based image processing. Therefore, the development of a method for qualitative and quantitative analysis of cashmere and wool would have great application value.

Recently, the application of metasurfaces in biosensors has attracted much attention. Metasurfaces are special artificially designed periodic sub−wavelength structures [13]. The optical properties of metasurfaces show many exotic phenomena that are not possible with natural materials, such as negative refraction [14], anomalous transmission [15,16,17], invisible cloaking [18], and perfect electromagnetic absorption [19,20,21,22]. Metasurfaces are different from conventional materials in that their main physical properties are determined by their unit structure in relation to the periodic distribution of arrays rather than by their own material properties. This allows one to artificially design the geometry of subwavelength cells to construct an electromagnetic response that can exhibit tunability in practical applications [23]. An electromagnetic response in metasurfaces called electromagnetically induced transparency (EIT) has attracted much interest from researchers. EIT originates from quantum physical systems, where a narrow and sharp window of transparency is formed in a wide absorption region due to phase cancellation interference between different excitation pathways [24]. When the broadband bright mode and the narrowband dark mode resonators are very close to each other in both the spatial and frequency domains, the phase cancellation interference between the broadband bright mode and narrowband dark mode resonators produces a narrow transparent window similar to that of the EIT [25]. Due to the narrow transparency window and strong local fields, EIT−like metasurface proves to be an ideal platform for detecting small changes in the surrounding media environment. Electromagnetically induced transparency also causes a large change in the transmission phase of electromagnetic waves through the transmission window created by the designed metamaterials and an increase in the group delay, leading to slow light properties, which can enhance some of the nonlinear effects [26,27]. The spectral response of EIT−like metasurfaces has been experimentally verified in the microwave, terahertz, and optical frequency ranges [28,29,30,31,32,33,34]. Since the electromagnetic response of biological macromolecules is in the terahertz band, the detection of wool and cashmere is more sensitive in the terahertz band as well.

This study introduces a terahertz EIT−like metasurface biosensor for the accurate qualitative and quantitative detection of wool and cashmere fibers. The periodic unit structure of the metasurface consists of four rotationally symmetric split rings (QSRR) and two cross−arranged metal secants, which form toroidal dipoles and electric dipoles, respectively. This configuration enables the effective sensing area to be greatly improved. By adjusting the size, a transparent window is formed at the frequency of 1.45 THz. The theoretical analysis indicates that the metasurface has a sensitivity of up to 342 GHz/RIU (RIU, refractive index unit) with a 20 μm thick analyte, and a Q factor up to 32.9. Cashmere and wool fiber, as a solution, were tested with the metasurface biosensor under a terahertz time−domain spectrometer (THz−TDS), and the experimental results demonstrated that frequency shifts ΔF were 246 GHz and 282 GHz, respectively, as the concentration of the cashmere and wool fiber solution changed from 1.0 mg/mL to 0.04 mg/mL. Moreover, it is evident that the amplitude and resonance frequency of the transmission spectra of the cashmere and wool fibers exhibit significant differences at the same concentration, thereby enabling the direct differentiation between two fibers. The proposed terahertz EIT−like metasurface represents a novel approach for achieving high sensitivity, accuracy, and trace detection of cashmere and wool, which is of paramount importance to the cashmere industry.

## 2. Methods

### 2.1. Terahertz Metasurface Sensing Scheme for the Detection of Cashmere and Wool

**Figure 1:**
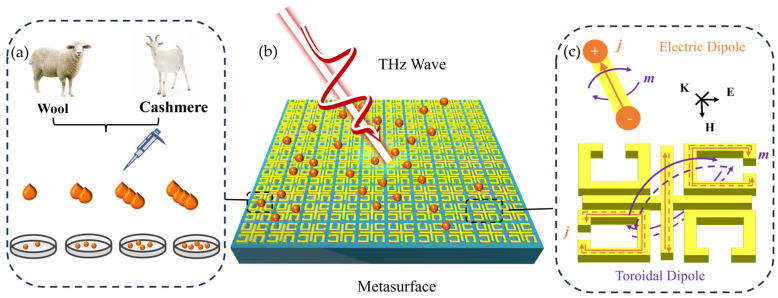
Schematics of the sensing strategy based on the proposed metasurface biosensor. (**a**) Cashmere and wool dissolution solution with a concentration gradient. (**b**) Terahertz time−domain spectroscopy (THz−TDS) captures the terahertz transmission spectra of the ETI−like metasurface spin−coated with the cashmere and wool solution (of the same thickness). (**c**) The electromagnetic mode of structure unit: the metal secant flowing in the direction of the excitation light polarization can be excited to form the electric dipole’s current; the two split rings on the diagonal can then generate the toroidal dipole’s circulating current (j, red arrow).

The terahertz metasurface sensing strategy is shown in Figure 1. The cashmere and wool dissolution solution, with a concentration gradient as shown in Figure 1a, was added on our proposed metasurface biosensor. The specific dissolution method of cashmere and wool is described in detail in the experimental section. Figure 1b illustrates the metasurface biosensor coated with the cashmere/wool solution under terahertz wave irradiation. The experimental procedure entails spin−coating the same thickness of cashmere/wool solution onto the metasurface, and then measuring the transmission spectra that would be collected by the THz−TDS. As illustrated in Figure 1c, the metal cut wire of the unit structure oriented in the direction of the excitation light polarization can be stimulated to form an electric dipole current; and a toroidal dipole can be excited by the circulating currents (j, red arrow) in the two split rings on the diagonal, which thus produces a magnetic dipole (m, purple arrow) with a similar radiation pattern in the far field. The interference in radiation patterns of the electric and toroidal dipoles excites the electromagnetically induced transparent window.

### 2.2. Metasurface Design

**Figure 2:**
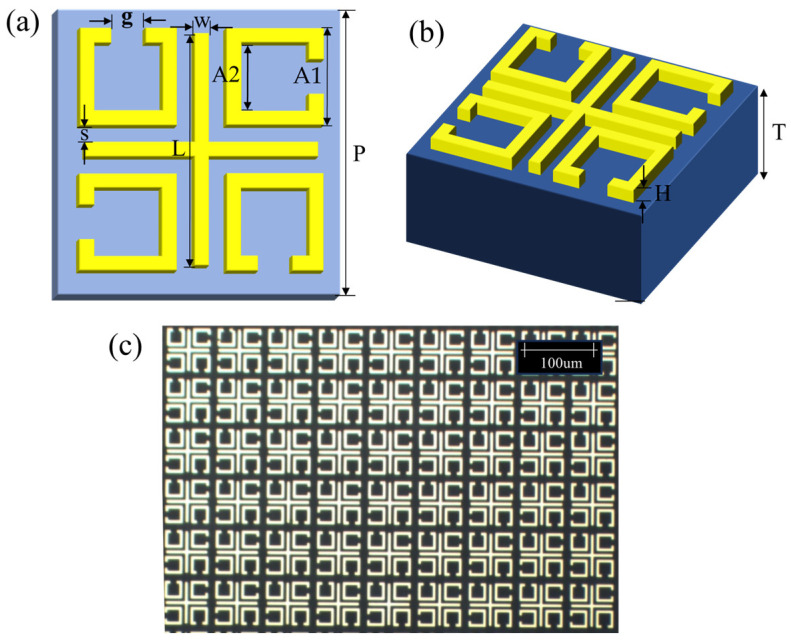
(**a**) Top view and (**b**) side view of the designed unit QSRRC structure: the square period P = 74 μm, the cross−arranged cut wires with L = 62 μm and W = 4 μm, the ring with A1 = 26 μm, A2 = 18 μm, the central split g = 9 μm, and the spacing S = 3 μm. The thickness of the substrate and gold layers are T = 100 μm and H = 0.15 μm, respectively. (**c**) Optical microscope image of the terahertz EIT−like metasurface.

To study the resonance spectral response of the EIT−−like metasurfaces and perform modal analysis, full−−wave numerical simulations were carried out using the commercial software, CST2022 Microwave Studio. In the simulation, the THz wave was vertically incident on the surface of the sensor, while the unit cell extended along the x and y directions, and an open boundary condition parallel to the x–y plane was set above the unit cell. The excitation light field was a plane wave propagating along the z−axis that was polarized in the x or y direction. The quadruple rotationally symmetric split−ring resonator and cross−arranged metal cut wire combination structure (QSRRC) has been designed as shown in Figure 2a. The geometric dimensions of the designed meta−atom are as follows: the square period P is set as 74 μm; two cross−arranged metal cut wires with L = 62 μm and W = 4 μm; four rotationally symmetric split rings with A1 = 26 μm, A2 = 18 μm, and the central split g = 9 μm; and the spacing S = 3 μm. The substrate and gold layer thicknesses were set as T = 100 μm and h = 0.15 μm, respectively. The conductivity of gold was 4.561 × 10^7^ s/m.

### 2.3. Fabrication of Metasurface Sensor

Periodic arrays were prepared on 100 μm thick, 5 × 5 mm^2^ sized quartz glass (ε = 3.85) substrates using maskless lithography, followed by high temperature evaporation of the 150 nm Au layers. Figure 2c presents an optical microscope image of the prepared EIT−like metasurface, observed in both the x and y directions. The entire physical size of the metal array was 7.6 mm × 7.6 mm.

### 2.4. Cashmere and Wool Sample Preparation and Terahertz Spectroscopic Measurement

The terahertz transmission spectra of the biosensor samples were analyzed using a commercially available THz−TDS (Advantest TAS7400TS) with a spectral resolution of 7.6 GHz. The effective frequency range was 0.1 to 4.0 THz. The biosensor was placed in a sample holder in the THz−TDS, perpendicular to the connection line between the radiation emitter and the detector. The terahertz transmission spectra of the biosensor were then coated with the cashmere/wool fiber lysate and further investigated at an ambient temperature of 15 °C and a relative humidity of less than 5%. The spectra measured in the time domain were converted to frequency domain data using the Fourier transform technique. The transmission values were normalized using air as a reference.

Levulinic acid (LA) and 1,5-diazabicyclo [4.3.0]-5-nonene (DBN,98%) were procured from Aladdin Commerce Reagent Co. The cashmere/wool underwent purification using a Soxhlet apparatus, employing a solvent mixture of acetone and ethanol (50%/50%) for a duration of 48 h to eliminate fats and other impurities. Subsequently, the purified cashmere/wool was washed with deionized water and dried at a temperature of 100 °C for 24 h before conducting the dissolution experiments. To obtain the [DBNH][Lev], 15.4 g of LA and 14.0 g of DBN were added to a single 100 mL double−necked glass vial. Subsequently, the mixture was stirred for a duration of five minutes in an ice bath [33]. Following this, the [DBNH][Lev] PILs were supplemented with 1 g of cashmere and wool fibers of varying mass ratios (100/0, 80/20, 60/40, 20/80, 0/100). To achieve a uniform dissolution of cashmere/wool, the mixture was subjected to mechanical stirring in an oil bath at a temperature of 120 °C for a duration of one hour.

## 3. Results and Discussion

### 3.1. Modes Analysis

**Figure 3:**
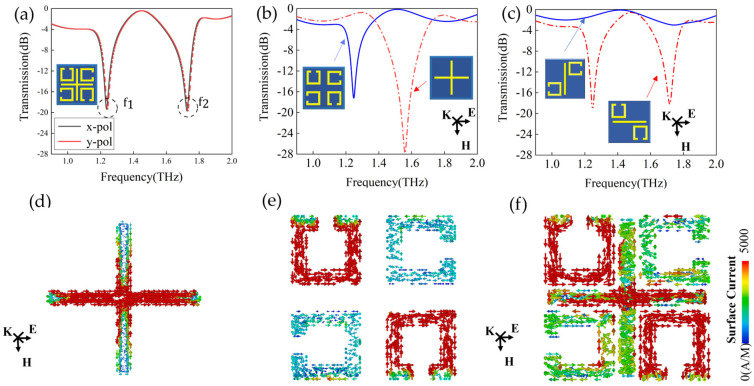
(**a**) Transmission spectrum simulation of the EIT−like metasurface under x− and y−polarized incident waves. (**b**) Simulated transmission spectrum of light mode (red line) and dark mode (blue line). (**c**) Transmission spectra of simulated split−ring resonators (SRR) and cut wires (CW) parallel and perpendicular to the electric field direction. The direction of the terahertz wave **k**, electric field **E**, and magnetic field **H** are perpendicular to each other. (**d**) Bright mode, (**e**) dark mode, and (**f**) surface current distribution of the entire metasurface at 1.24 THz.

Figure 3a–c demonstrate the simulated transmission spectra of the proposed metasurface biosensor consisting of the CW and the four SRR. The EIT−like resonance was excited by either x− or y−polarized ultrashort laser pulse. As shown in Figure 3a, the transmission spectra under the x− and y−polarization incident waves exhibit significant overlap, with two distinct resonance dips evident at f1 and f2. This observation indicates that the proposed metasurface possesses polarization independence due to its four−fold rotational symmetry. As such, a quick response time and direct measurements can be achieved in real bio−detection situations. Under the x−polarized laser pulses, both the CW and SRR can be individually excited with the resonance frequencies of 1.24 and 1.72 THz, respectively (shown as Figure 3b). The surface currents oscillate on the CW and SRR as shown in Figure 3d and 3e, respectively; the CW in the direction of polarization and the SRR on the diagonal produce a strong resonance with a high Q factor, while the CW in the non−polarized direction and SRR on the diagonal have little interaction with the incident wave (shown as Figure 3c). As shown in Figure 3d,e, regarding the resonance frequencies of f1, the surface currents oscillate on the CW along with the x−polarization, which acts as a bright mode; while two of the SRRs located on the diagonal can be excited with a distinct circulating current in the opposite direction, which then induces a magnetic dipole, leading to the formation of a dark mode characterized by small radiative loss. Due to the similarity in indirect excitation strength between the SRR and the CW and a phase difference of π [28], the bright and dark resonance modes interfere with each other and result in the transparent window at the frequency of 1.45 THz.

**Figure 4:**
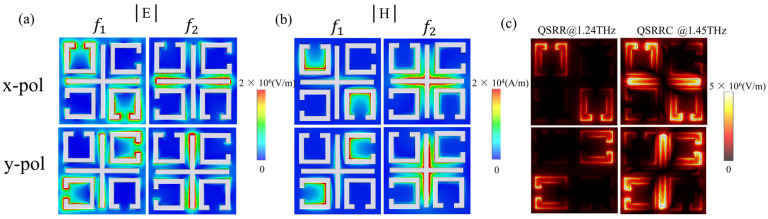
(**a**) Near−electric−field distribution of x− and y−polarized excitation at the f1 and f2 resonance frequencies of the QSRRC metasurface. (**b**) Near−magnetic−field distribution of x− and y− polarized excitation at the f1 and f2 resonance frequencies of the QSRRC metasurface. (**c**) Near−electric−field distribution of x− and y−polarized excitation of QSRR at f = 1.24 THz and QSRRC at the transparent window f = 1.45 THz.

By placing the monitor at the resonance frequencies of f1 and f2, we can capture the x−polarized and y−polarized surface electric and magnetic field energy distributions at f1 and f2, as shown in Figure 4a. In the toroidal dipole resonance at the f1 frequency, the electric field enhancement is mainly concentrated at the opening of the resonance ring, while the magnetic field enhancement at the f2 frequency is in the two metal secant accessories at the x and y polarization areas, as shown in Figure 4b. Local electromagnetic field excitation enhances the electromagnetic induction transparency (EIT) effect, significantly improving sensor detection accuracy.

The relationship between the resonance frequency and capacitance can be stated as f = 1⁄((2π × (LC)^0.5), which is widely recognized. As the size of the split area in the SRR expands, the capacitance also grows. Additionally, the width of the gap between the SRR and the CW further contributes to the growth in capacitance. The capacitance, C, is equal to the sum of the dispersed capacitance, Cdc, and the plate capacitance, Cpc [35]. The equation for capacitance (Cpc) is given by Cpc = εS/4πkd, where ε is the dielectric constant between the two plates, k is the electrostatic force constant, d is the distance between the plates, and S is the area of the two plates. A greater clearance between the wires results in an increased area of the sensing zone. The magnitude of the change in the resonance frequency of the sensor due to the variation in the dielectric constant of the analyte is directly proportional to the magnitude of the change in the capacitance (C).

The near−electric−field distribution diagram of the quadruple rotationally symmetric split−ring resonator (QSRR) at f = 1.24 THz is shown in Figure 4c. Similar to the electric field enhancement of the metasurface biosensors with conventional dipole resonance, which is limited to the SRR opening, we found that the QSRRC at 1.45THz showed electric field enhancement near both the opening and the metal cut wire. The QSRRC metasurface not only has a wider detection area, but also a stronger constrained electromagnetic energy. Therefore, it can be determined that the EIT−like resonance has a larger range of photoactive regions than the dipole resonance, which greatly expands the spatial range of the interaction between light and matter, meaning that the effective sensing area is greatly increased. The calculated total sensing area of QSRRC is about 1075% higher than that of QSRR. In the majority of cases, the sample molecules of the biosensor structure are dissolved in the reagent and subsequently deposited on the sensor surface. The wide−area ultra−strong light active region formed by the QSRRC metasurface will greatly improve the probability of capturing trace molecules by biosensors and promote the coupling of light field energy and sample molecules.

### 3.2. Factors That Influence Sensitivity of the Metasurface Sensor Covered with Analytes

**Figure 5:**
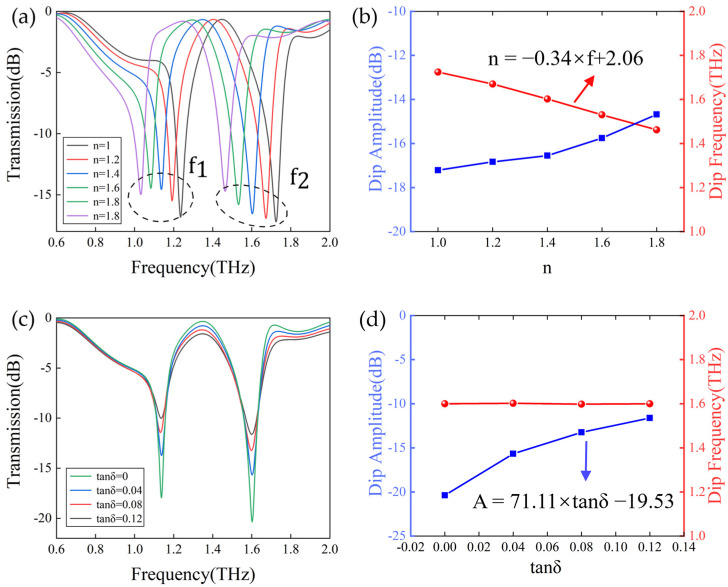
(**a**) Simulated transmission spectrum of a biosensor covered with a 20 μm thick analyte with a refractive index increased from 1.0 to 1.8. (**b**) Frequency shift (peak transmission) versus refractive index of the analyte. (**c**) Dependence of amplitude on analyte loss tangent increased from 0 to 0.12. (**d**) Frequency shift (peak transmission) versus loss tangent of analyte.

In order to further investigate the sensing properties of the metasurface, we first evaluated the transmission spectra of the metasurface covered with analytes with different refractive indices (n) and dielectric losses (tanδ) by numerical simulations. In the simulation, we followed the principle of controlling variables and, firstly, added a layer of analyte with a thickness of h = 20 μm on the metasurface, while keeping its analyte dielectric loss tanδ at 0.03. Upon increasing the refractive index n of the analyte from 1 to 1.8, the sensitivity was evaluated in terms of the magnitude and frequency of resonance inclination, as illustrated in Figure 5a.Two distinct resonance dips were evident at f1 and f2.This figure demonstrates that both resonance peaks exhibit near−peak transmittance. Both resonance peaks exhibit a linear redshift with a decrease in amplitude, but the resonance peak f2 shows a better change in resonance inclination, frequency linearity, and higher Q factor compared to f1. This makes the resonance peak f2 more suitable for the accurate and reliable identification and quantification of substances. Figure 5b illustrates the linear−fitting relationship between the frequency at resonance peak f2 and the increasing amplitude expressed by the equation: f = −0.34 × n + 2.0612. The slope of the curve, the sensitivity (Sn) is as high as 342 GHz/RIU (refractive index unit). Consequently, the varying composition and concentration of the analyte result in a change in the equivalent refractive index, which, in turn, affects the frequency shift of the resonance peaks.

Furthermore, the dielectric loss in the analyte also leads to a significant response in the transmission spectra of the sensor. The dielectric loss factor (tanδ) is often regarded as a quantitative parameter describing the dielectric loss. As illustrated in Figure 5c, an increase in the resonance inclination from 0 to 0.12 is accompanied by a rise in tanδ from 0 to 0.12, while maintaining n = 1.4. This results in a gradual decline in the Q factor. Furthermore, the dielectric loss exerts a relatively minor influence on the resonance frequency, which remains almost constant. Figure 5d shows the relationship between resonance inclination and tanδ, and the fitted curve is represented by A = 71.77 × tanδ − 19.53. Consequently, the difference in the compositional components of the analytes may result in varying dielectric losses, which can be distinguished by the change in amplitude as observed in the transmission spectra.

We also list the performance parameters of this work and recently reported metasurface biosensors. The sensitive radioactivity aptitude of this sensor is compared with some reported structures, demonstrating the superiority of this design. The comparison results are shown in Table 1.

### 3.3. Application of Metasurface Sensors for Terahertz Spectroscopic Detection of Cashmere and Wool

**Figure 6:**
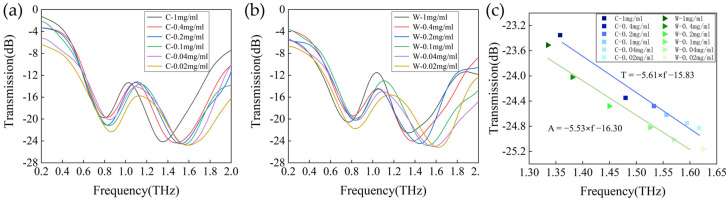
Transmission spectra of the proposed EIT−like biosensor for the (**a**) cashmere and (**b**) wool fibers at 1 mg/mL–0.04 mg/mL concentration. (**c**) Linear fits of resonance frequency shifts and peak amplitudes as a function of concentration for the cashmere and wool fibers in the concentration range of 1 mg/mL to 0.04 mg/mL.

To confirm the ability of the developed metasurface sensor to differentiate between cashmere and wool, we conducted measurements of the terahertz transmission spectra of the sensors. These measurements were taken for various concentrations of cashmere and wool as shown in Figure 6a,b. The attachment of both fibers resulted in varying degrees of increasing amplitude and redshifts in the resonance frequency, which are consistent with our prediction for the analytes. The concentrations of wool fibers were 0.02, 0.04, 0.1, 0.2, and 1 mg/mL, and the the resonance dip shifts were 103 GHz, 133 GHz, 194 GHz, 271 GHz, 340 GHz, and 385 GHz, respectively. The concentrations of cashmere fibers were elevated at 0.02, 0.04, 0.1, 0.2, and 1 mg/mL, while the resonance dip shifts were 134 GHz, 141 GHz, 164 GHz, 202 GHz, 248 GHz, and 370 GHz, respectively. This discrepancy may be attributed to the distinct physical properties of the two fibers, such as their varying sizes or dielectric constants.

Further analysis of the data characterizing the operating frequency and inclination of cashmere/wool fibers at different concentrations with varying fiber concentrations in the terahertz band reveal that although the amplitude and frequency of the resonance inclination angle are not strictly linear with respect to the fiber concentration, they exhibit a consistent overall trend. Specifically, with the increase in fiber concentration, the amplitude and frequency of the resonance inclination show a certain degree of increase or decrease. This trend provides an important basis for distinguishing different fibers in this paper. In order to visualize this result, a linear fit of the resonance frequency shift and peak amplitude with concentration is shown in Figure 6c. The resonance inclination amplitude and frequency distributions of the cashmere fibers were represented by blue squares, with the fitting equation T = −5.61 × f − 15.83, while the corresponding data of wool fibers were represented by green triangles, with the fitting equation T = −5.53 × f − 16.30. By comparing and analyzing the distribution characteristics of these two regions, it is possible to discern the differences between cashmere and wool fibers in terms of resonance inclination amplitude and frequency. This enables the qualitative identification of the sample fibers, provided that the resonance inclination amplitude and frequency data for the fiber samples are available.

**Figure 7:**
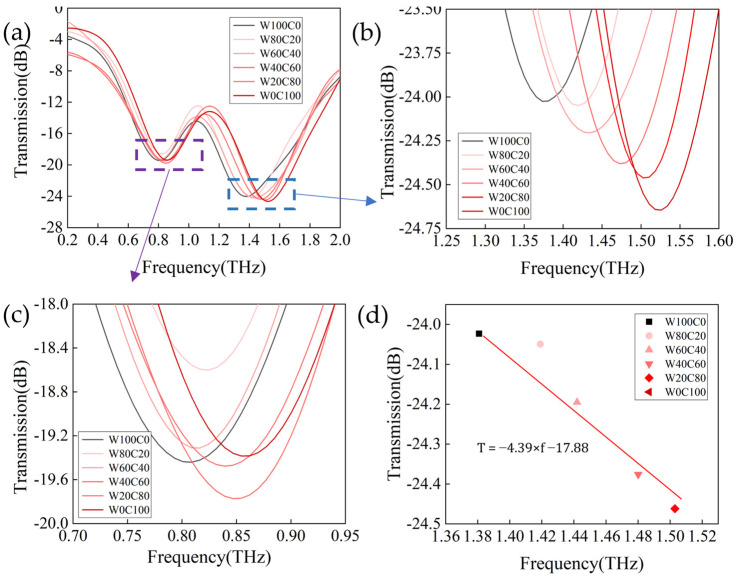
(**a**) Transmission spectra of cashmere/wool blended fibers at the same concentration of 0.2 mg/mL. Zoomed view of the blue (**b**) and purple (**c**) boxes. (**d**) Linear−fitting plot of resonance frequency shift and amplitude values of cashmere/wool mixed fibers with cashmere content at 0.2 mg/mL concentration.

For quantitative analysis, the terahertz transmission spectra of the blended cashmere/wool fibers at different mass ratios (100/0, 80/20, 60/40, 20/80, 0/100) at a concentration of 0.2 mg/mL were collected. As shown in Figure 7a, with the increase in cashmere content, both resonance peaks f1 and f2 undergo a decrease in amplitude and a redshift in resonance frequency. Figure 7b,c show the thumbnail view of the resonance peaks f1 and f2, respectively, and we found that the amplitude change at f1 is irregular and its inclination is relatively flat. More importantly, the cashmere content is increased from 0% to 100%, and its maximal frequency shift at f1 is 45 GHz, which means it has low detection accuracy.

Fortunately, we found a sharper resonance inclination and obvious amplitude and frequency changes at the resonance peak f2, with a maximum frequency shift of 137 GHz. We plotted a two−dimensional linear fit of amplitude transmittance and resonance frequency at f2 for the different cashmere content levels. The diagram shows a consistent and predictable line, as shown in Figure 7d. This discovery enables us to accurately determine the content of cashmere in cashmere/wool blended fabrics and achieve quantitative analysis of cashmere/wool.

## 4. Conclusions

In this study, an EIT−like metasurface sensor for the quantitative and qualitative analysis of cashmere/wool fibers was designed and fabricated. This EIT−like metasurface consists of four SRR and CW that can form a transparent window at a resonance frequency of 1.45 terahertz. The proposed EIT−like metasurface has a high sensitivity of 342 GHz/RIU and a high Q factor of 32. A special EIT−like design can establish a large ultra−intense light response region, thereby enhancing the capture of molecules by the metasurface sensors and facilitating the efficient transfer of light field energy to sample molecules. This enables the demonstration of a biosensing technique for the quantitative and qualitative analysis of cashmere/wool fibers. In addition, metasurface sensors have strong rotational stability, which facilitates potential advances in the detection of actual animal fibers in textiles due to their specific polarization independence.

The high sensitivity of the sensor to the refractive index of the analyte allowed for the addition of different concentrations of the cashmere/wool fiber solution to the metasurface sensor. Transmission spectroscopy measurements revealed that the two types of fibers differed in their resonance inclination in terms of amplitude and frequency. This allowed for a qualitative analysis of the fibers. The fiber types are identified by comparing spectral features; therefore, by adding different mass ratios of the cashmere/wool fiber solution, it was found that the amplitude and frequency of the resonance inclination were regularly weakened and redshifted. This demonstrated the possibility of predicting the trend of the resonance inclination with the mass ratio of cashmere, enabling the quantitative detection of cashmere. The proposed metasurface sensor can provide a powerful tool to directly differentiate between analyzed fiber types and concentrations in a single measurement. Its label−free, polarization−independent, and high−sensitivity characteristics are of great significance in the cashmere industry.

## Figures and Tables

**Table 1:** The comparison of the sensitivity of sensors based on metasurfaces.

Resonance Type	Sensitivity (GHz/RIU)	Detection Target	Metal and Reference
EIT−like resonance	131.05	Cancer Cells	Gold [36]
Electric dipole Resonance	76.5	carcinoembryonic antigen	Al [37]
Dipole resonance	200	amyloid β aggregates	Gold [38]
Fano resonance	240	Protein	Cu [39]
Electric dipole Resonance	85	Protein	Al [40]
EIT−like resonance	342	cashmere/wool	Gold This work

## Data Availability

No new data were created or analyzed in this study. Data sharing is not applicable to this article.
